# S-15176 Difumarate Salt Can Impair Mitochondrial Function through Inhibition of the Respiratory Complex III and Permeabilization of the Inner Mitochondrial Membrane

**DOI:** 10.3390/biology11030380

**Published:** 2022-02-27

**Authors:** Natalia V. Belosludtseva, Vlada S. Starinets, Alena A. Semenova, Anastasia D. Igoshkina, Mikhail V. Dubinin, Konstantin N. Belosludtsev

**Affiliations:** 1 Laboratory of Mitochondrial Transport, Institute of Theoretical and Experimental Biophysics, Russian Academy of Sciences, Institutskaya 3, 142290 Pushchino, Moscow Region, Russia; vlastar@list.ru (V.S.S.); dubinin1989@gmail.com (M.V.D.); bekonik@gmail.com (K.N.B.); 2 Department of Biochemistry, Cell Biology and Microbiology, Mari State University, pl. Lenina 1, 424001 Yoshkar-Ola, Mari El, Russia; sem_al.ru@mail.ru (A.A.S.); anastasi.igoshkina@yandex.ru (A.D.I.)

**Keywords:** S-15176, mitochondria, mitochondrial membrane potential, mitochondrial respiration, electron transport chain, mitochondrial membrane permeabilization

## Abstract

**Simple Summary:**

The metabolic agent S-15176 difumarate salt belongs to a potent class of drugs called partial fatty acid oxidation inhibitors. The possible therapeutic effects of this agent have been widely investigated in models of oxidative damage to different tissues, but the underlying mechanisms are currently unclear and require further research. In the present study, we aimed to investigate in more detail the effect of S-15176 on the mitochondria, power stations inside the cell, and pivotal regulators of many cellular processes. We found that S-15176 affected the key indicators of the function of rat liver and thymocyte mitochondria at the concentrations reached in target tissues. S-15176 disturbed the catalytic function of enzyme complex III of the mitochondrial electron transfer system and induced nonspecific membrane permeability, which was related to the dissipation of the mitochondrial membrane potential and a decline in ATP production. These findings lead us to predict that S-15176 can induce mitochondrial dysfunction in mammal tissues and organs that are most vulnerable to chemical toxicity or exposed to higher concentrations of the drug.

**Abstract:**

S-15176 difumarate salt, a derivative of the anti-ischemic metabolic drug trimetazidine, has been intensively studied for its impact on cellular metabolism in animal models of ischemia-reperfusion injury of the liver, heart, spinal cord, and other organs. Despite evidence of some reduction in oxidative damage to cells, the results of therapy with S-15176 have been mostly disappointing, possibly because of the lack of data on its underlying mechanisms. Here, we aimed to investigate in more detail the role of complexes I-IV of the electron transport chain and membrane permeability transition in mitochondrial toxicity associated with S-15176. Using rat thymocyte and liver mitochondria, we demonstrated that: (1) acute exposure to S-15176 (10 to 50 μM) dose-dependently decreased the mitochondrial membrane potential; (2) S-15176 suppressed the ADP-stimulated (State 3) and uncoupled (State 3U_DNP_) respiration of mitochondria energized with succinate or malate/glutamate, but not ascorbate/TMPD, and increased the resting respiration (State 4) when using all the substrate combinations; (3) S-15176 directly inhibited the activity of the respiratory complex III; (4) low doses of S-15176 diminished the rate of H_2_O_2_ production by mitochondria; (5) at concentrations of above 30 μM, S-15176 reduced calcium retention capacity and contributed to mitochondrial membrane permeabilization. Taken together, these findings suggest that S-15176 at tissue concentrations reached in animals can impair mitochondrial function through suppression of the cytochrome *bc*1 complex and an increase in the nonspecific membrane permeability.

## 1. Introduction

Trimetazidine (TMZ), the derivative of piperazine, is an anti-ischemic metabolic agent of a potent class of drugs called partial inhibitors of fatty acid oxidation (pFOX). Meta-analyses of clinical studies have proved the therapeutic effect of TMZ against stable angina. Nowadays, TMZ is included in the guidelines of the European Society of Cardiology for the management of stable angina pectoris and stable coronary artery disease [1,2,3]. Some studies have demonstrated that TMZ has a cytoprotective effect in models of ischemic damage to the kidney and liver [4,5], as well as in several metabolic pathologies, including diabetes mellitus [6,7,8]. The beneficial effect of TMZ on ischemic tissues is mainly attributed to its inhibitory action on the long-chain 3-ketoacyl-coenzyme A thiolase, resulting in inhibition of the beta-oxidation pathway of free fatty acids [9,10,11]. TMZ can also increase the activity of pyruvate dehydrogenase, which is the rate-limiting enzyme of glucose oxidation under aerobic conditions [10,11,12]. This, by switching cellular metabolism from the oxidation of free fatty acids towards the utilization of carbohydrates, would improve the efficiency of ATP synthesis for a given amount of oxygen molecules consumed, thereby maintaining the energy supply in hypoxia [12]. Taken together, TMZ can shift the energy substrate metabolism, enhancing glucose metabolism and decreasing oxygen consumption. This may be accompanied by decreased ROS production, a limited increase in intracellular acidosis, and reduced accumulation of cytosolic calcium. All of these events are related to the mitochondria, which are likely to have several targets for this drug. Recent studies suggest that TMZ inhibits the opening of the mitochondrial permeability transition (MPT) pore, contributing to protection against dysfunction of mitochondria and cell death in response to ischemia [13,14,15].

S-15176 (N-{(3,5-di-*tert*-butyl4-hydroxy-1-thiophenyl)}-3-propyl-N′-(2,3,4-trimethoxybenzyl) piperazine) difumarate salt is a derivative of TMZ (Figure S1), but its therapeutic efficacy and mechanisms of action have not yet been fully understood. The drug can act on several targets in the mitochondria. In particular, S-15176 has been reported to inhibit the rate-limiting enzyme of the beta-oxidation cycle carnitine palmitoyltransferase I (CPT-1) [16], suppress the formation of the MPT pore and the cyclosporin A-insensitive mitochondrial pore, and prevent free radical-induced toxicity [17,18]. These effects are suggested to underlie the protective action of S-15176 against mitochondrial and cellular dysfunction in ischemia–reperfusion injury of liver and myocardium tissues, traumatic spinal cord injury, and experimental diabetes mellitus [19,20,21,22,23].

Some studies on isolated mitochondria revealed that, in addition to the above effects, S-15176 could induce mitochondrial uncoupling and reverse the activity of the ATP synthase, leading to the hydrolysis of ATP molecules [17,24]. However, the mechanism of these processes remains unknown. It was suggested that the S-15176-induced decrease in the mitochondrial membrane potential proceed through a disruption of the function of the respiratory chain complexes. One can assume that treatment with S-15176 may lead to mitochondrial dysfunction, impaired energy metabolism, ion dyshomeostasis, and, ultimately, cell death in tissues that are most vulnerable to the toxic action of chemicals or exposed to higher doses of the agent. Therefore, a study of the role of the mitochondrial membrane potential and mitochondrial enzyme complexes of the respiratory chain in S-15176-induced toxicity is needed, as well as a study of the involvement of mitochondrial ROS production and permeability transition, which could potentially provide possibilities for interventions on the adverse effects caused by this agent.

Based on these considerations, we used rat thymocytes and isolated rat liver mitochondria as model objects to examine the effect of S-15176 at different concentrations on the mitochondrial membrane potential and mitochondrial respiratory capacities when using different combinations of respiratory substrates. To determine which complex(es) of the electron transfer system is (are) affected by S-15176, we estimated the enzymatic activity of complexes I-IV in the presence of this agent. We then evaluated its effects on the generation of mitochondrial ROS and Ca^2+^-induced permeability transition as possible mechanisms of the drug-associated mitotoxicity. Our results indicated that acute exposure to S-15176 resulted in a higher dissipation of the membrane potential of the organelles energized with succinate or malate/glutamate, but not ascorbate/TMPD. In addition, S-15176 difumarate salt inhibited complex I- and complex II-linked ADP-stimulated respiration but had no effect on CIV-linked ADP-stimulated respiration, which could be due to its direct inhibitory action on enzyme complex III of the mitochondrial electron transfer system. We also found that low doses of S-15176 diminished the production of H_2_O_2_ and increased the calcium retention capacity index, while at concentrations above 30 μM, S-15176 contributed to mitochondrial membrane permeabilization.

## 2. Materials and Methods

### 2.1. Chemicals

S-15176 difumarate salt was purchased from Sigma-Aldrich (St. Louis, MO, USA). The stock solution of the drug was prepared in dimethylsulfoxide (DMSO). In control experiments, an equivalent volume of the solvent was used. The final concentration of DMSO in a cuvette did not exceed 0.1%. All other chemicals used were supplied by Sigma-Aldrich (St. Louis, MO, USA), except where indicated.

### 2.2. Preparation of Rat Thymocytes and Measurement of Mitochondrial Membrane Potential

The thymus from two male Wistar albino rats (90–110 g) was used to isolate thymocytes according to [25]. The medium used for isolation and incubation of thymocytes contained 145 mM NaCl, 5.6 mM KCl, 10 mM glucose, 8 mM Mops/KOH, pH 7.4. Control samples contained 80–90% live cells. All measurements were performed within the first three hours after cell isolation to maintain similar cell viability and mitochondrial potential. Cell parameters were assessed by flow cytometry using the Muse cell analyzer (Luminex, Austin, TX, USA). The Muse MitoPotential Kit (MCH100110, Luminex, Austin, TX, USA) was used to evaluate the mitochondrial membrane potential of isolated thymocytes.

### 2.3. Isolation of Mitochondria from Rat Liver

For isolation of mitochondria, the liver of male Wistar albino rats was homogenized in a medium that included 70 mM sucrose, 210 mM mannitol, 1 mM EDTA, and 10 mM Hepes/KOH, pH 7.4. Subsequent differential centrifugation procedures were performed according to the conventional protocols as previously described [26]. Next, centrifugations used the same medium, but 100 μM EGTA replaced EDTA. The resulted suspension contained 60–70 mg of mitochondrial protein per mL (determined by the Bradford method).

### 2.4. Measurements of Mitochondrial Bioenergetics

The rate of O_2_ consumption by mitochondrial samples was estimated using Oxygraph Plus (Hansatech Instruments Ltd., Pentney, King’s Lynn, UK) [26]. The reaction medium included 130 mM KCl, 5 mM NaH_2_PO_4_, and 10 mM Hepes/KOH, pH 7.4. The following combinations of substrates and reagents were used: 2.5 mM potassium glutamate + 2.5 mM potassium malate, 5 mM potassium succinate + 1 µM rotenone, 5 mM ascorbic acid + 0.2 mM *N*,*N*,*N′*,*N′*-tetramethyl-*p*-phenylenediamine (TMPD) + 1 µM rotenone. These conditions are necessary for studying the oxygen-consuming capacity of the organelles through complexes I, II, and IV of the respiratory chain, respectively. To determine the rates of the phosphorylating respiration (State 3) and uncoupled respiration (State 3U_DNP_) of mitochondria, 200 µM ADP and 50 µM 2,4-dinitrophenol (DNP) were added sequentially. The solutions of S-15176 difumarate salt or DMSO were added to the mitochondrial suspension at a concentration of 1 mg of mitochondrial protein per mL 1 min before the start of measurements.

Mitochondrial membrane potential (Δψ) was evaluated by the distribution of the penetrating ion tetraphenylphosphonium bromide (TPP) in the suspension of the organelles, which was assessed with a TPP-sensitive electrode (Nico-Analyte Ltd., Moscow, Russia) in a temperature-controlled chamber. The electrode was calibrated with known levels of TPP^+^ at the start of each experiment. The reaction medium included 210 mM mannitol, 70 mM sucrose, 10 μM EGTA, 10 mM Hepes/KOH, and pH 7.4. TPP^+^ was added to the incubation medium at a concentration of 1.5 μM. The following substrates and reagents were used: 2.5 mM potassium glutamate + 2.5 mM potassium malate, 5 mM potassium succinate + 1 µM rotenone, and 5 mM ascorbate + 0.2 mM TMPD + 1 µM rotenone. The measurements required 1 mg of mitochondrial protein per mL. In parallel, the changes in ΔΨ were analyzed using the fluorescent dye safranine O (λ_ex_ = 520 nm; λ_em_ = 580 nm) and the Panorama Fluorat-02 spectrofluorometer (Lumex, Saint Petersburg, Russia), as described earlier [25].

The S-15176 effect on the activity of the mitochondrial respiratory complexes (I, II, III, IV) was estimated according to the protocols [27,28] using a Multiskan GO plate spectrophotometer (Thermo Fisher Scientific, Waltham, MA, USA). The measurements were performed on disrupted mitochondria subjected to three cycles of freezing/thawing at −20/+30 °C in the 10 mM Tris/HCl buffer, pH 7.6. The suspension of 50 μg/mL mitochondrial protein was used in each measurement. The activity of complex I was estimated at 25 °C and 600 nm by the 2,6-dichlorophenolindophenol (DCIP) reduction in the presence of disrupted mitochondria [27]. The medium was based on 25 mM potassium phosphate, pH 7.5, supplemented with 60 µM DCIP, 70 µM decylubiquinone, 3.5 mg/mL BSA, 10 µg/mL antimycin A. The redox reaction was started with 100 µM NADH. The activity of complex II was evaluated at 37 °C and 600 nm by the DCIP reduction in the presence of disrupted mitochondria fueled with succinate [28]. The medium was based on 25 mM potassium phosphate, pH 7.5, and supplemented with 300 µM NaCN, 1 mg/mL BSA, and 20 mM succinate. The redox reaction was started with 50 µM decylubiquinone. The activity of complex III was measured at 25 °C and 550 nm by the cytochrome *c* reduction in the presence of disrupted mitochondria [25]. The medium was based on 25 mM potassium phosphate, pH 7.5, and supplemented with 100 µM EDTA, 75 µM of oxidized cytochrome *c,* and 0.025% (vol/vol) Tween-20. The reaction was started by 100 µM decylubiquinol. The activity of complex IV was estimated at 25 °C and 550 nm by oxidation of reduced form of cytochrome *c* [28]. The medium based on 50 mM potassium phosphate, pH 7.0, was supplemented with 60 µM initially reduced (using sodium dithionite) cytochrome *c*. The reaction was started by disrupted mitochondria.

### 2.5. Assessment of H_2_O_2_ Production by Liver Mitochondria

The fluorescent probe Amplex Red (λ_ex_ = 560 nm; λ_em_ = 590 nm) and a plate reader Tecan Spark 10 M were used to register mitochondrial H_2_O_2_ generation [26]. In this case, 0.15 mg of mitochondrial protein was incubated in the buffer containing 70 mM sucrose, 210 mM mannitol, 1 mM KH_2_PO_4_, 5 mM succinate, 10 µM Amplex Red, 1 µM rotenone, 10 µM EGTA, horseradish peroxidase (HRP 1 a.u./mL), 10 mM Hepes/KOH, and pH 7.4. The kinetics of H_2_O_2_ generation was recorded for three minutes. The amount of the resulting H_2_O_2_ was calculated from the calibration curve. A stock solution of hydrogen peroxide was prepared on the day of the experiment; its concentration was determined using the molar absorption coefficient E_240_ = 43.6 M^−1^ × cm^−1^.

### 2.6. Quantification of Mitochondrial Calcium Retention Capacity Index

The concentration of Ca^2+^ was estimated in a temperature-controlled chamber using an ion-selective electrode (Nico-Analyte Ltd., Moscow, Russia) connected to a computerized recording system Record 4 (Pushchino, Russia) [26]. The suspension of 1.0–1.2 mg of mitochondrial protein was incubated in the buffer that included 70 mM sucrose, 210 mM mannitol, 1 mM KH_2_PO_4_, 5 mM succinate, 1 μM rotenone, 10 μM EGTA, 10 mM Hepes/KOH, and pH 7.4. Then, CaCl_2_ (10 μM) was sequentially added to the sample cuvette until a massive Ca^2+^ release from the mitochondria began. The total Ca^2+^ added to mitochondria before the MPT pore opening was interpreted as the mitochondrial Ca^2+^ retention capacity and expressed as nmol Ca^2+^/mg of mitochondrial protein.

### 2.7. Mitochondrial Swelling Assay

The mitochondrial swelling process was evaluated as a decrease in absorbance at 540 nm (*A*_540_) using an Ocean Optics USB-2000 spectrophotometer (Ocean Insight, Orlando, FL, USA) [26]. The swelling buffer included 210 mM mannitol, 70 mM sucrose, 5 mM succinate, 1 μM rotenone, 10 μM EGTA, 10 mM Hepes/KOH, and pH 7.4.

### 2.8. Statistical Analysis

Data obtained were analyzed using GraphPad Prism 7.0 (SPSS Inc., Chicago, IL, USA). The data were presented as the means ± SEM of 3–6 independent experiments. Mann–Whitney *U* tests were used for statistical processing, and *p*-value < 0.05 was interpreted as statistically significant.

## 3. Results

### 3.1. S-15176 Difumarate Salt Promotes Mitochondrial Depolarization in Rat Thymocytes

**Figure 1:**
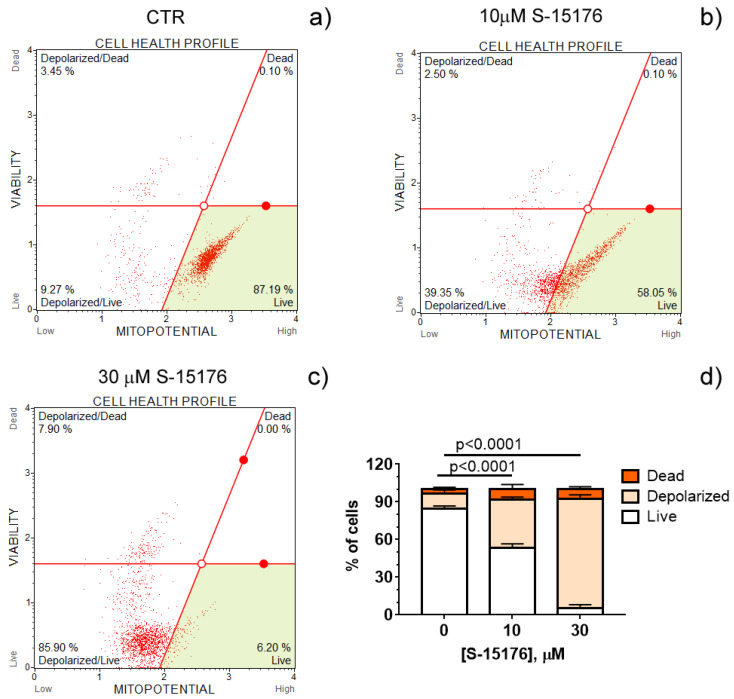
S-15176 difumarate salt induces the depolarization of the mitochondrial membrane in rat thymocytes. Mitochondrial membrane potential (MitoPotential) was assessed with the Muse Cell Analyzer using the Muse MitoPotential kit. Cells were treated with 0 μM S-15176 (**a**), 10 μM S-15176 (**b**), or 30 μM S-15176 (**c**) for 30 min. Typical profile plots are presented. Panel (**d**) shows the ratio (%) of living, depolarized, and dead rat thymocytes in the presence of S-15176 at different concentrations. In control experiments (CTR, 0 µM S-15176), an equivalent volume of the solvent (0.1% DMSO) was used. Data represent the mean ± SEM (*n* = 4).

Increasing evidence suggests that S-15176 difumarate salt acts on several targets in the mitochondria [16,17,18,24]. In this study, we evaluated whether this drug affects the key indicators of mitochondrial activity. The mitochondrial membrane potential is known to be the main index of mitochondrial function since it reflects the processes of electron transfer and oxidative phosphorylation. Our results demonstrated that S-15176 difumarate salt could initiate mitochondrial depolarization in rat thymocytes. As shown in Figure 1, preincubation of rat thymocytes with S-15176 at concentrations of 10 and 30 µM for 30 min resulted in a decline in the mitochondrial membrane potential in a dose-dependent manner. Importantly, the addition of S-15176 at a concentration of 30 µM led to complete mitochondrial depolarization in the whole cell population.

### 3.2. S-15176 Inhibits ADP- and 2,4-Dinitrophenol- Stimulated Mitochondrial Respiration Due to Suppression of the Enzymatic Activity of the Respiratory Complex III

To elucidate the mechanism of mitochondrial depolarization caused by S-15176, we investigated the effect of the agent on the efficiency of oxidative phosphorylation (OXPHOS) and the enzymatic activity of the individual OXPHOS complexes I–IV in isolated mitochondria.

**Figure 2:**
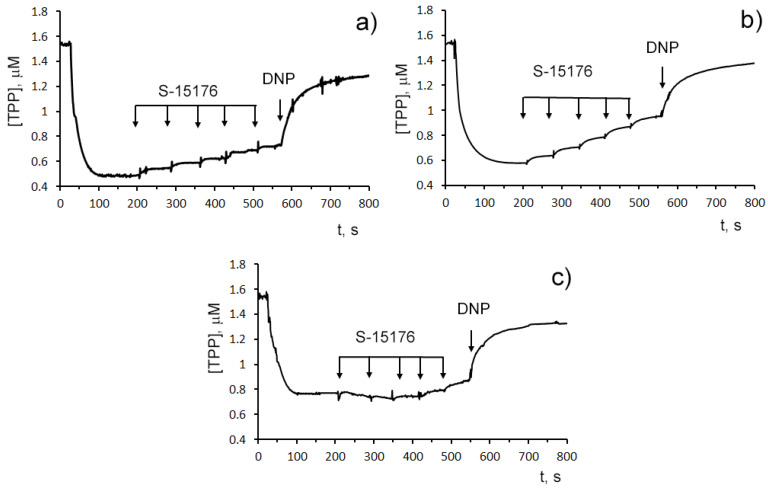
Sequential addition of 10 μM S-15176 gradually decreases the membrane potential of isolated rat liver mitochondria energized by the complex I substrates (2.5 mM potassium glutamate + 2.5 mM malate) (**a**), or the complex II substrate (5 mM potassium succinate in the presence of 1 µM rotenone) (**b**), but not by the complex IV substrates (5 mM ascorbic acid + 0.2 mM TMPD) (**c**). Mitochondrial membrane potential was estimated by the distribution of tetraphenylphosphonium bromide (TPP^+^) with an ion-sensitive electrode. Additions: 10 µM S-15176 (five pulse additions), 50 µM DNP. Typical traces are shown (*n* = 6).

The influence of S-15176 on the mitochondrial bioenergetics was assessed by the rate of oxygen consumption by rat liver mitochondria in the main metabolic states using different combinations of respiration substrates. Table 1 demonstrates the effect of S-15176 on the respiration rates of mitochondria oxidizing the substrates of complex I (2.5 mM glutamate and 2.5 mM malate) or complex II (5 mM succinate in the presence of 1 μM rotenone). One can see that when using both of these combinations of substrates, S-15176 difumarate salt stimulated mitochondrial respiration under resting conditions (States 4 and 2) in a dose-dependent manner (Table 1). Moreover, S-15176 dose-dependently suppressed the rates of mitochondrial respiration in the presence of ADP (State 3) or the uncoupler 2,4-dinitrophenol (DNP) (State 3U_DNP_). In parallel, the respiratory control ratio (RCR) (State 3/State 4), which is directly related to the OXPHOS coupling efficiency, was reduced by 2.1 and 1.8 times in the presence of 30 µM S-15176 when using the substrates of complex I and complex II, respectively. Furthermore, the successive addition of 10 μM S-15176 pulses to the mitochondrial suspension resulted in a gradual decrease in the membrane potential of mitochondria energized by complex I- or complex II-linked respiratory substrates (Figure 2). The latter is consistent with the literature data that S-15176 can display an uncoupling activity in rat liver mitochondria [24].

By contrast, when the substrates needed for the complex IV activity (ascorbate and TMPD) were used, S-15176 at concentrations of 10 and 30 µM had no effect on the rate of oxygen consumption by mitochondria in States 3 and 3UDNP (Table 1). In this case, the addition of 30 µM S-15176 led to a decrease in the RCR by only 9%, which was due to a slight stimulation of the resting state respiration (State 4) with the agent. Importantly, the S-15176-induced decline in the membrane potential of rat liver mitochondria incubated with ascorbate + TMPD was observed only at high concentrations of this agent (above 40 µM), and it was significantly less pronounced compared with that when using malate + glutamate or succinate as substrates (Figure 2). It should be noted that similar results were obtained when using the fluorescent dye safranin O as an indicator of the mitochondrial membrane potential (Figure S2). These findings suggest that, in addition to the proton conductance activity, S-15176 has the ability to directly impair certain components of the mitochondrial electron transfer system.

Indeed, a spectrophotometrical assay of the activity of OXPHOS enzymes revealed that 30 μM S-15176 decreased the activity of complex III by 1.6 times but did not affect complexes I, II, and IV in isolated mitochondria (Table 2).

### 3.3. S-15176 Inhibits H_2_O_2_ Production by Rat Liver Mitochondria

**Figure 3:**
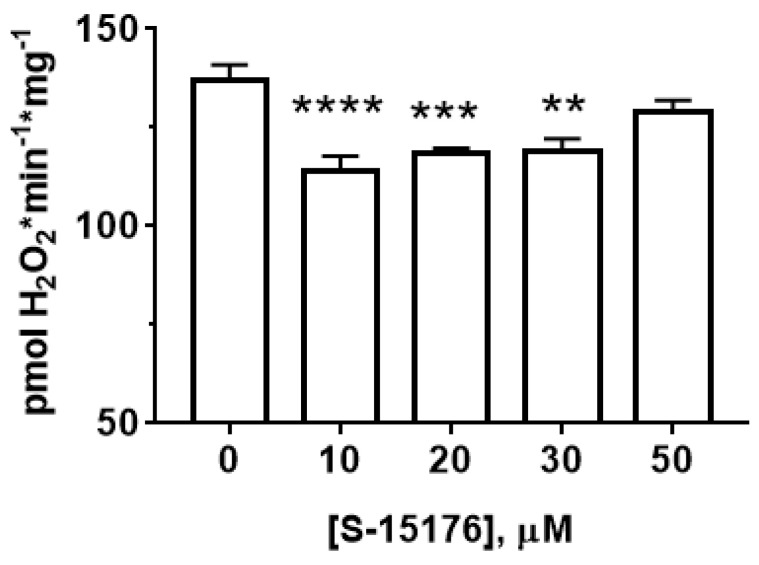
Effect of S-15176 on the rate of H_2_O_2_ production by rat liver mitochondria. Data represent the mean ± SEM (*n* = 5). ** *p* < 0.01, *** *p* < 0.005, and **** *p* < 0.001.

Next, we measured the effect of S-15176 on the rate of H_2_O_2_ generation in rat liver mitochondria (Figure 3). Our results showed that at concentrations of 10 and 30 µM, S-15176 significantly inhibited the rate of H_2_O_2_ production by the mitochondria. However, this effect was canceled with a further increase in the concentration of the drug. The addition of 50 µM S-15176 to the mitochondria returned the rate of H_2_O_2_ formation to the control level.

### 3.4. S-15176 at Low Concentrations Suppress the Opening of the Ca^2+^-Dependent MPT Pore and, at High Concentrations, the Agent Itself Contributes to the Permeabilization of the Mitochondrial Membrane

**Figure 4:**
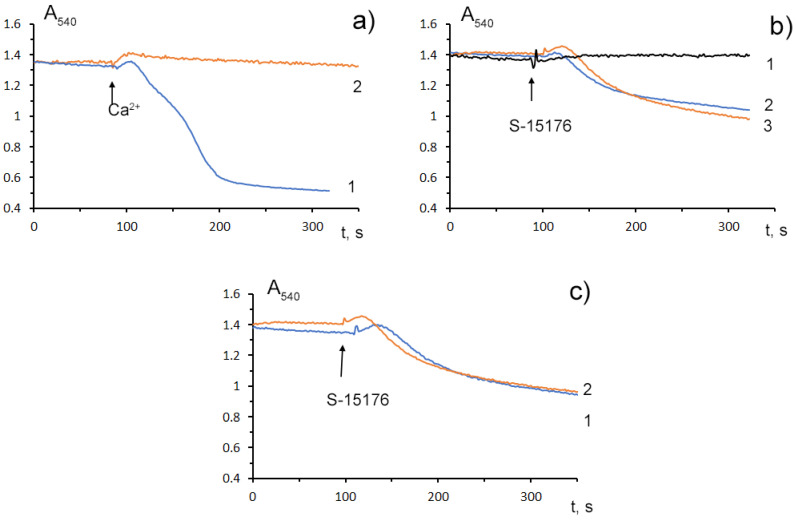
Swelling assay of rat liver mitochondria (0.35 mg/mL) treated with S-15176 difumarate salt; (**a**) Preincubation of mitochondria with 10 μM S-15176 for 1 min (2) blocks mitochondrial swelling induced by 50 μM Ca^2+^ in the presence of 1 mM P_i_ (1); (**b**) S-15176 by itself induces mitochondrial swelling when its concentration is increased from 10 µM (1) to 30 µM (2) or 50 µM (3); (**c**) Pretreatment of rat liver mitochondria with 1 μM cyclosporin A (2) does not affect mitochondrial swelling induced by 30 μM S-15176 (1). Typical traces are shown (*n* = 5).

Mitochondrial Ca^2+^ overload is one of the key mechanisms involved in the opening of the MPT pore and mitochondrial damage [29]. Some studies demonstrated that S-15176 could inhibit the formation of the Ca^2+^-dependent MPT pore in mitochondria. We observed that 10 μM S-15176 suppressed the high-amplitude swelling of rat liver mitochondria induced by 50 μM Ca^2+^ in the presence of 1 mM P_i_ (Figure 4a). Alternatively, 30 μM S-15176 by itself induced a decrease in the optical density of the mitochondrial suspension (Figure 4b), indicating the beginning of the swelling process. It should be noted that the amplitude of mitochondrial swelling induced by S-15176 was less than that after the MPT induction. No further increase in the amplitude of S-15176-induced mitochondrial swelling was observed when using the concentrations of S-15176 above 30 μM. It is important to note that the addition of the selective inhibitor of the MPT pore opening cyclosporin A did not affect the S-15176-induced swelling of mitochondria (Figure 4c), supporting the conclusion that S-15176 in high concentrations could promote mitochondrial membrane permeabilization through an MPT-independent mechanism.

**Figure 5:**
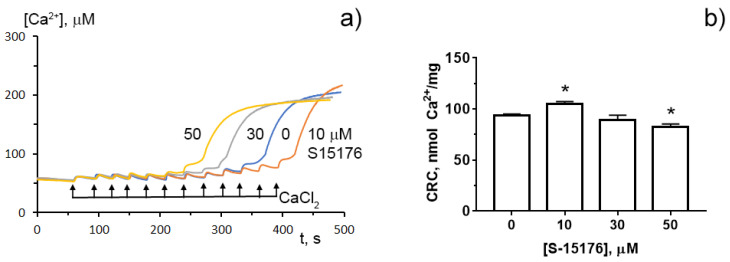
Typical changes in the external (Ca^2+^) upon the successive addition of 10 μM CaCl_2_ pulses to the suspension of rat liver mitochondria in the presence of S-15176 (10, 30, and 50 µM) or 0.1% DMSO (0 µM S-15176); (**a**) The action of S-15176 difumarate salt on the calcium retention capacity index of rat liver mitochondria; (**b**) The index reflects maximum Ca^2+^ overload of mitochondria that occurs immediately before the MTP pore opening. Data represent the mean ± SEM (*n* = 5). * *p* < 0.05.

To determine whether S-15176 can reduce the susceptibility of mitochondria to the formation of the classical MPT pore, we next assessed the calcium retention capacity index, which reflects the maximum overload with Ca^2+^ that occurs immediately before the MTP pore formation. Our results demonstrated that 10 µM S-15176 increased this indicator, while 50 μM S-15176 stimulated the Ca^2+^-induced MPT pore opening (Figure 5). These observations allow us to conclude that S-15176 has a dual effect on mitochondrial Ca^2+^ overload depending on the concentration used.

## 4. Discussion

The derivative of TMZ S-15176 difumarate salt has been found to act as a regulator of intracellular metabolism and can interact with several molecular targets in mitochondria [16,17,18,24] that are known to be key to the supply of most of the ATP molecules used by cells, cell signaling, ion homeostasis, and the events leading up to apoptosis and necrosis [30,31,32,33]. Some studies have shown that targeting mitochondria with S-15176 has a therapeutic effect in the oxidative stress-related pathologies of different organs and tissues [19,20,21]. It was reported that the S-15176-induced suppression of excessive ROS generation by mitochondria and downregulation of mitochondrial proteins that are essential for the formation of the MPT pore complex could contribute to the preservation of the functions of both mitochondria and cells upon ischemia–reperfusion damage to tissues in S-15176-treated animals [18,24]. The anti-ischemic effect of this agent can be mediated by shifting from the oxidation of fatty acids to the oxidation of glucose, with IC50 for the mitochondrial carnitine palmitoyltransferase in liver homogenate being 50.8 μM [16].

In this work, we provide evidence that, along with the modulation of cellular metabolism, S-15176 difumarate salt at concentrations of 30 µM and above can contribute to mitochondrial damage through direct suppression of complex III of the respiratory chain and an increase in the nonspecific permeability of the inner membrane. We used rat thymocytes and rat liver mitochondria to demonstrate that acute exposure to this metabolic agent can induce mitochondrial dysfunction by similar mechanisms. A growing body of evidence has suggested mitochondrial dysfunction as a mechanism for drug-induced toxicity in general [34] and also for the liver and thymus [35,36]. It is known that the thymus, as the place of T cell generation, is very vulnerable to the harmful effects of drugs. The higher vulnerability of the thymus gland to toxic agents makes this organ a marker of metabolism disorders and early functional defects in the immune system [35]. At the same time, the liver is considered the principal site of drug metabolism and is responsible for the selective uptake, concentration, processing, and excretion of most drugs that are introduced into the organism. Drug-induced inhibition of mitochondrial activity is thought to be the main mechanism whereby drugs can promote cellular injury and inflammation in the liver [36]. One can suggest that mitochondrial toxicity associated with S-15176 may cause the dysfunction of both organs and subsequent aggravation of pathologies.

Earlier, it was reported that S-15176 can exhibit protonophoric activity in the mitochondrial membranes [18,24]. The data obtained in this work are in line with these findings. We demonstrated that the administration of S-15176 to isolated rat liver mitochondria stimulated the oxygen consumption rate of the organelles under resting conditions (States 4 and 2) (Table 1), indicating a perturbation in the proton motive force created by the respiratory chain. One could speculate that the uncoupling action of S-15176 may underlie the antioxidant properties of this drug. Indeed, one can see that the addition of S-15176 at moderate concentrations to mitochondria suppressed the production of H_2_O_2_ by the organelles (Figure 3).

On the other hand, we observed that as early as 10 μM S-15176 caused a significant decrease in the mitochondrial membrane potential in rat thymocytes. Moreover, thymocytes treated with 30 μM S-15176 showed complete mitochondrial depolarization. Similarly, S-15176 (from 10 to 50 μM) substantially reduced the membrane potential of isolated mitochondria energized with the respiratory substrates of complex I or complex II. It is also important to note that 10 μM S-15176 decreased the ADP- and DNP-stimulated respiration of rat liver mitochondria when using complex I- or complex II-linked substrates. In this case, there was a significant decrease in the RCR. Contrariwise, 30 μM S-15176 reduced the RCR by only 9% when the substrates of complex IV ascorbate and TMPD were used. The S-15176-induced depolarization of isolated mitochondria energized by the complex IV-linked respiratory substrates was also insignificant.

Based on the data obtained, one can assume that S-15176 acts on the bioenergetic indicators of rat liver mitochondria through several mechanisms. The increase in State 4 respiration, which was observed in the presence of all the substrate combinations, could be associated with the uncoupling effect of S-15176 on mitochondria. The fact that the S-15176-induced decrease in State 3 and 3U_DNP_ respiration and, as a consequence, in the RCR when using substrates of complexes I and II, but not complex IV, allowed us to hypothesize that this agent can affect specific components of the electron transfer system. By assessing the enzyme activity of the respiratory complexes I-IV, we showed that S-15176 significantly suppressed the activity of the complex III of the respiratory chain, coenzyme Q: cytochrome c oxidoreductase.

Accumulating evidence suggests that S-15176 difumarate salt inhibits mitochondrial Ca^2+^-induced permeability transition by a mechanism independent of its antioxidant action [17]. It was found that the pretreatment of rat liver mitochondria with the drug suppressed the formation of both the classical cyclosporine A-sensitive MPT pore and the cyclosporine A-insensitive mitochondrial pore under the conditions of Ca^2+^ and ROS stress [17]. The data obtained in our work confirmed that S-15176 at low doses is able to suppress the opening of the Ca^2+^-induced MPT pore in mitochondria. We demonstrated that 10 μM S-15176 inhibited Ca^2+^-induced mitochondrial swelling and increased the mitochondrial calcium retention capacity index, indicating a decrease in the susceptibility of the organelles to the MPT pore induction. At concentrations of 30–50 μM, S-15176 reduced the ability of mitochondria to retain calcium ions, which suggests that high levels of the drug could attenuate the resistance of mitochondria to the opening of the MPT pore. Since the molecular structure of the MPT pore complex has not been fully established to date [29], it is not yet possible to find out with certainty which protein component of this complex the metabolic agent S-15176 may interact with.

When the concentration of S-15176 was increased to 30 μM, the drug itself caused a high-amplitude swelling of the organelles. Taking into account the fact that cotreatment with cyclosporin A, a selective blocker of the MPT pore, had no effect on this process, one can suggest that there were no specific interactions between S-15176 and the protein components of the pore complex. The mechanism of the acute impairment of membrane permeability by this amphiphilic compound is not precisely known but may be related to the accumulation of the drugs in the inner mitochondrial membrane and temporary disturbance of the lipid bilayer, as was previously observed upon the induction of cyclosporin A-independent permeability transition pore caused by long-chain free fatty acids [29,37].

## 5. Conclusions

S-15176 difumarate salt can interact with mitochondria and affect their function through the regulation of several molecular targets. As shown in the current investigation, despite the positive effect of low doses of S-15176 on some mitochondrial functions, acute exposure to this drug in concentrations over 30 µM can be toxic to the mitochondria and cells. Our in vitro studies showed that 30 µM S-15176 could decrease complex I and II-linked respiration due to direct inhibition of the activity of the mitochondrial respiratory complex III coenzyme Q:cytochrome c-oxidoreductase and promote cyclosporin A-insensitive mitochondrial swelling. These effects of S-15176, along with its known protonophoric activity, are likely to underlie the decline in the membrane potential, the driving force behind ATP production, which was observed in both rat thymocytes and isolated liver mitochondria. Altogether, these findings suggest that S-15176 at tissue concentrations reached in animals can cause mitochondrial dysfunction through similar mechanisms in organs that are most vulnerable to chemical toxicity or exposed to higher concentrations of the drug.

## Figures and Tables

**Table 1:** Effects of S-15176 difumarate salt on the respiration of rat liver mitochondria.

S-15176, μM	V Respiration, nmol O_2_ × min^−1^ × mg^−1^ Protein				
	State 2	State 3	State 4	State 3U_DNP_	RCR
Glutamate + malate					
0	3.1 ± 0.2	21.5 ± 1.1	3.5 ± 0.1	21.8 ± 1.0	5.9 ± 0.1
10	4.0 ± 0.3 *	18.1 ± 0.8 *	3.9 ± 0.2	17.9 ± 0.9 *	4.7 ± 0.1 ***
30	4.5 ± 0.3 **	16.0 ± 0.2 **	5.9 ± 0.1 **	15.9 ± 0.2 **	2.7 ± 0.1 ***
Succinate					
0	6.0 ± 0.3	32.8 ± 0.6	6.9 ± 0.2	47.2 ± 1.2	4.8 ± 0.1
10	7.7 ± 0.2 **	30.7 ± 0.6 *	8.4 ± 0.3 **	44.1 ± 0.8	3.7 ± 0.2 **
30	8.9 ± 0.2 **	29.3 ± 0.9 *	10.8 ± 0.3 **	40.9 ± 1.4 *	2.7 ± 0.1 ***
Ascorbate + TMPD					
0	25.4 ± 0.6	34.9 ± 1.0	23.7 ± 0.4	38.9 ± 1.3	1.5 ± 0.1
10	27.1 ± 0.8	35.3 ± 0.8	25.0 ± 0.5	37.7 ± 0.9	1.4 ± 0.1
30	29.0 ± 0.5 **	35.7 ± 0.5	26.7 ± 0.3 *	36.4 ± 1.1	1.3 ± 0.1 *

Oxygen consumption of mitochondria was fueled by 2.5 mM glutamate and 2.5 mM malate, 5 mM succinate (in the presence of 1 μM rotenone), and 5 mM ascorbate + 0.2 mM TMPD. Data represent the mean ±SEM (*n* = 5). * *p* < 0.05, ** *p* < 0.01, *** *p* < 0.001—differences between the control (with 0.1% DMSO, 0 µM S-15176) and the experiment (with 10 or 30 µM S-15176) were statistically significant.

**Table 2:** Effect of S-15176 difumarate salt (30 μM) on the activity of complexes (CI-IV) of the mitochondrial respiratory chain.

Values in % of Activity Compared with the Control (100%)			
CI	CII	CIII	CIV
102.7 ± 1.9	99.4 ± 3.2	63.3 ± 1.4 *	105.1 ± 3.7

In the absence of S-15176 (control, 0.1% DMSO), the activities of complexes I, II, III, and IV were 385 ± 5, 435 ± 4, 679 ± 9, 467 ± 17 nmol*min−1*mg^−1^, respectively. The activity values in the absence of S-15176 were taken as 100%. Data represent the mean ±SEM (*n* = 3). * Differences between the control (0.1% DMSO) and the experiment (30 µM S-15176) were statistically significant (*p* < 0.05).

## Data Availability

The data presented in this study are available on request from the corresponding author.

## Supplementary Materials

The following supporting information can be downloaded at: https://www.mdpi.com/article/10.3390/biology11030380/s1, Figure S1. Structural formula of S-15176 N-[(3,5-Di-tert-butyl-4-hydroxy-1-thiophenyl)]-3-propyl-N′-(2,3,4-trimethoxybenzyl)piperazine difumarate salt; Figure S2. Representative recordings of changes in the fluorescence intensity of safranine O, which reveal S-15176-induced depolarization of isolated rat liver mitochondria energized by the complex I substrates (2.5 mM potassium glutamate + 2.5 mM malate) (a), the complex II substrate (5 mM potassium succinate in the presence of 1 µM rotenone) (b), or the complex IV substrates (5 mM ascorbic acid + 0.2 mM TMPD) (c).
