# Supplementary material for: S-15176 Difumarate Salt Can Impair Mitochondrial Function through Inhibition of the Respiratory Complex III and Permeabilization of the Inner Mitochondrial Membrane
Source: Biology (Basel). 2022 Feb 27;11(3):380. doi: 10.3390/biology11030380 (PMC8945000; doi:10.3390/biology11030380)
Supplement: Supplementary file 1 [file biology-11-00380-s001.zip › biology-1606545-supplementary.pdf]

Supplementary Materials

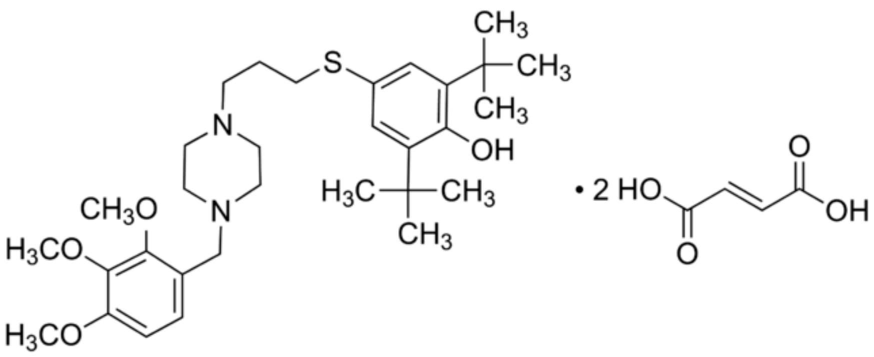

Figure S1. Structural formula of S-15176 N-[(3,5-Di-tert-butyl-4-hydroxy-1-thiophenyl)]-3-propyl-N′-(2,3,4-trimethoxybenzyl)piperazine difumarate salt.

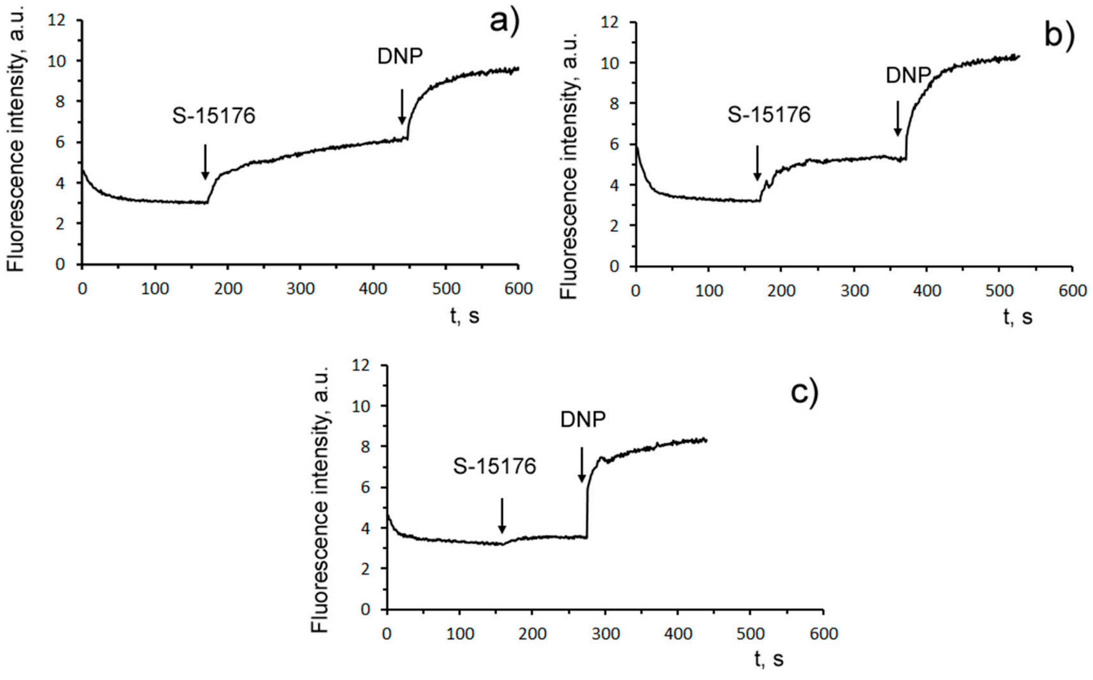

Figure S2. Representative recordings of changes in the fluorescence intensity of safranine O, which reveal S-15176-induced depolarization of isolated rat liver mitochondria energized by the complex I substrates (2.5 mM potassium glutamate + 2.5 mM malate) (**a**), the complex II substrate (5 mM potassium succinate in the presence of 1 μM rotenone) (**b**), or the complex IV substrates (5 mM ascorbic acid + 0.2 mM TMPD) (**c**). Additions: 30 μM S-15176, 50 μM DNP. The mitochondrial membrane potential was evaluated with safranine O as described in the Materials and Methods section. Typical traces are shown (*n* = 5).
